# E-Cigarette Use among Smokers with Serious Mental Illness

**DOI:** 10.1371/journal.pone.0113013

**Published:** 2014-11-24

**Authors:** Judith J. Prochaska, Rachel A. Grana

**Affiliations:** 1 Stanford Prevention Research Center, Department of Medicine, Stanford University, Stanford, California, United States of America; 2 Center for Tobacco Control Research and Education, Cardiovascular Research Institute, University of California San Francisco, San Francisco, California, United States of America; Centre for Addiction and Mental Health, Canada

## Abstract

**Background:**

We examined electronic cigarette (EC) use, correlates of use, and associated changes in smoking behavior among smokers with serious mental illness in a clinical trial.

**Methods:**

Adult smokers were recruited during acute psychiatric hospitalization (N = 956, 73% enrollment among approached smokers) in the San Francisco Bay Area between 2009–2013. At baseline, participants averaged 17 (SD = 10) cigarettes per day for 19 (SD = 14) years; 24% intended to quit smoking in the next month. Analyses examined frequency and correlates of EC use reported over the 18-month trial and changes in smoking behavior by EC use status.

**Findings:**

EC use was 11% overall, and by year of enrollment, increased from 0% in 2009 to 25% in 2013. In multiple logistic regression, the likelihood of EC use was significantly greater with each additional year of recruitment, for those aged 18–26, and for those in the preparation versus precontemplation stage of change, and unlikely among Hispanic participants. EC use was unrelated to gender, psychiatric diagnosis, and measures of tobacco dependence at baseline. Further, over the 18-month trial, EC use was not associated with changes in smoking status or, among continued smokers, with reductions in cigarettes per day.

**Interpretation:**

Within a clinical trial with smokers with serious mental illness, EC use increased over time, particularly among younger adults and those intending to quit tobacco. EC use was unrelated to changes in smoking. The findings are of clinical interest and warrant further study.

## Introduction

Tobacco is a major public health concern [1], and smokers with serious mental illness (SMI) are increasingly gaining recognition as a disparity group and priority population [2], [3]. SMI has been defined as having a diagnosable psychiatric disorder with functional impairment that substantially interferes with or limits one or more major activities [4]. Smoking is endemic among individuals with SMI, with a prevalence of 40% to 60%[5], [6] compared to 19% in the general population. Smoking prevalence among persons with SMI has declined more slowly relative to the general population suggesting national tobacco control policies have not reached this group as effectively [7]. As an example, psychiatric units remain uniquely exempted from federal hospital smoking bans that took effect back in 1992 [8].

The consequences are significant. Individuals with SMI are dying on average 25 years prematurely; leading causes of death are chronic illnesses, most tobacco-related [9]. Tobacco smoke induces the metabolism of a number of psychiatric medications leading to sub-therapeutic blood levels and the need for higher doses [10]. As more private and public settings ban secondhand smoke (e.g., worksites, restaurants, parks), tobacco addiction serves to further isolate and stigmatize smokers with SMI. Further, the financial costs of tobacco are high, in one study, accounting for nearly a third of monthly incomes among smokers with schizophrenia [11].

Most smokers with SMI want to quit, and tobacco treatment trials have demonstrated treatment efficacy in samples of smokers diagnosed with major depression, posttraumatic stress disorder, schizophrenia, and alcohol or illicit-drug use disorders [12]–[17]. Evidence supports the efficacy of computer-assisted interventions tailored to readiness to quit, cognitive-behavioral interventions, and the use of nicotine replacement therapy (NRT) [14], [16], bupropion [13], and varenicline [18], [19]. Importantly, interventions that have helped patients quit smoking have not adversely affected their cognitive functioning, mental health recovery, or long-term sobriety [15]–[17], [20], [21].

Not all smokers, however, are ready to quit; and even among those who do quit, the stark reality is the majority will relapse. Long-term quit rates hover around 25%, true for smokers regardless of SMI diagnosis [22]. For smokers not sufficiently aided by existing evidence-based treatments, some will seek commercialized alternatives. One novel alternative to smoking that is gaining a great deal of attention, with widespread advertising and an expanding market, is the electronic cigarette, or e-cigarette (EC).

### The Emerging EC Market

EC are battery-powered devices that generate an aerosol for inhalation typically containing nicotine. Vigorous debate in the public sphere and scientific literature concerns the potential for EC as a “safer” alternative to tobacco cigarettes for smokers unable or unwilling to quit or for use as a smoking cessation aid [23], [24]. Awareness and use among both adults and teens has been rising rapidly in the past 3 years globally, particularly in Europe and the US [25]–[28]. In the general US adult population, EC awareness increased from 16% in 2009 to 58% in 2011, and use increased from 1% to 6% in the same timeframe [29]. The majority of users were current or former smokers, with dual use of EC and tobacco cigarettes being common. Attraction of EC included use in settings where conventional cigarettes are banned and perceptions that EC may aid cessation or are a healthier alternative to traditional cigarettes. One analyst projects EC will eclipse the conventional cigarette market in the US within the next decade [30]. Popular media has conjectured, without empirical evidence, that e-cigarettes may be good for mental health with suggested use in locked psychiatric wards [31].

Compared to NRT, proponents argue EC are more appealing to smokers because they mimic cigarettes in appearance, method of inhalation, and production of smoke-like aerosol. A lab-based crossover study with 40 smokers reported that EC shared a similar pharmacokinetic profile to the NRT inhaler; both EC and the inhaler reduced desire to smoke following overnight abstinence relative to placebo; the EC, however, was rated as more pleasant to use [32]. In terms of exposure risks, analysis of 12 first generation brand ECs found varying levels of toxic and carcinogenic compounds in the aerosol across brands, about 9 to 450 times lower than cigarette smoke, and toxicants in some brands, on some measures, were comparable to the NRT inhaler [33]. A study of nonsmokers' secondhand exposures found similar levels of cotinine (a metabolite of nicotine) for EC aerosol versus tobacco cigarette smoke [34].

Research on EC is limited but growing, with most studies to date being descriptive. Only three published trials have reported on efficacy of EC for smoking cessation [35]–[37]. A proof of concept study with 40 smokers not intending to quit retained 68% (27/40) at 6-months: 5 continued exclusive cigarette smoking, 13 were using both EC and cigarettes, and 9 of the initial 40 (22.5%) stopped using tobacco cigarettes entirely [35]. In a randomized trial with 300 smokers not intending to quit in the next month, 12-month quit rates were 4% for non-nicotine EC, 9% for nicotine EC tapered 7.2 to 5.4 mg, and 13% for 7.2 mg nicotine EC, not significantly different by condition [36]. In a randomized trial with 657 smokers interested in quitting, verified 6-month prolonged abstinence was 7% for 16 mg nicotine EC, 6% for 21 mg NRT patch, and 4% for placebo EC, not significantly different by condition [37]. Adherence was greater for EC than patches (78% vs. 46% at 1 month) though confounded in the study by differences in distribution (i.e., EC mailed directly to participants, while NRT-vouchers provided for redeeming at local pharmacies). EC users reported liking the products' tactile, cigarette-like qualities, sensory familiarity, taste, perceived health benefits, ease of use, and absence of cigarette odor.

To date, the only publication of EC use in smokers with SMI is a feasibility study with 14 smokers with schizophrenia not intending to quit [38]. At week 52, 50% (7 of 14) reported smoking 50% fewer cigarettes per day and 14% (2 of 14) were smoke-free; schizophrenia symptoms did not increase with smoking reduction or cessation. Prevalence of EC experimentation and use among smokers with SMI has not been reported.

The current study examined use of EC among smokers with SMI participating in an ongoing randomized tobacco treatment clinical trial. Relative to usual care, the trial is evaluating brief and extended smoking cessation treatment initiated in acute inpatient psychiatry settings and continued post-hospitalization. Despite the study's provision of combination NRT (i.e., patch plus gum or lozenge) for up to 6-months duration, EC use among participants appears to be on the rise. To add to the nascent literature on EC, we aimed to identify (1) correlates of EC use in our diverse psychiatric sample and (2) associated changes in smoking behavior over time.

## Methods

### Design

Data were analyzed from a three-group randomized clinical trial with N = 956 smokers with SMI (clinicaltrials.gov registration #NCT00968513). The three study groups were usual care (n = 134), brief treatment (n = 414), and extended treatment (n = 408). Randomization was blocked on hospital unit, cigarettes per day (>15), and stage of change (precontemplation, contemplation, preparation). The treatment groups received a computer-assisted intervention tailored to readiness to quit, a stage-tailored manual, brief stage-tailored on-unit counseling session with study staff, and study-provided combination NRT available following hospitalization. NRT was provided for 3 months in the brief treatment arm and 6-months in the extended treatment arm. The extended group also was offered 10 sessions of cognitive behavioral cessation counseling. Treatment was initiated during the smoke-free hospitalization and continued post-hospitalization with follow-up at months 3, 6, 12, and 18. The institutional review boards at Stanford University, the University of California, San Francisco, and Alta Bates Medical Center approved the study procedures; participants provided written informed consent.

### Sample

Participants were daily smokers of 5 or more cigarettes/day (due to provision of NRT in the treatment groups) recruited between November 2009 and September 2013 from one of seven 100% smoke-free acute care units (median length of stay <7 days) at four psychiatric hospitals in the San Francisco Bay Area. Eligibility criteria were purposefully broad to obtain a representative sample. Exclusions were non-English speaking; medical contraindications to NRT use (pregnancy, recent myocardial infarction); and lack of capacity to consent as determined by a 3-item screener of study purpose, risks, and benefits [39]. Use of alternative tobacco products such as EC was not an exclusion criterion. Intent to quit smoking was not required to participate, as the counseling approach was tailored to readiness to change. Among eligible patients approached for recruitment, 73% enrolled in the study.

### Measures

When the trial started in 2009, EC was not prevalent in the marketplace, and measures of EC were uncommon in research or clinical practice. Though EC use was not asked directly, an open-ended question at baseline and each follow-up assessed “all forms of tobacco use.” At the time of this trial, EC were considered by the US courts to be tobacco products. Over time, this item has captured reports of EC use among study participants at baseline and follow-up assessments. This broad question has been retained in the study to detect all emerging tobacco product use and to allow comparability of data over the five years of the trial.

To describe the sample, we assessed gender, ethnicity, race, age, and employment status. Tobacco use measures included years of smoking, usual number of cigarettes smoked prior to hospital admission, and time to first cigarette (TTFC) in the morning, dichotomized as within 30 minutes or longer. Stages of Change Scale assessed readiness to quit smoking with defined stages of precontemplation, not intending to quit smoking in the next 6 months; contemplation, intending to quit within 6 months; and preparation, planning to quit within 30 days with at least one 24-hr past-year quit attempt [40]. Psychiatric diagnosis was determined with the Mini-International Neuropsychiatric Interview Screener (MINI) with major DSM-IV diagnostic categories of unipolar depression, bipolar depression, psychotic disorders, and alcohol or illicit drug use disorders [41]. Measures of psychiatric symptom severity and mental health functioning were the Behavior and Symptom Identification Scale summary score (BASIS-24)[42] and the 12-item Short Form mental health composite scale (SF-12) [43].

### Analyses

To describe the sample and use of EC, we calculated means and frequencies. To address our study aims, in step 1, we ran a logistic regression model to identify study factors, demographic, psychiatric, and tobacco-related variables associated with EC use. We categorized smoking level at 1 pack per day or more and time to first cigarette as within 30 minutes. In step 2, we compared EC users and nonusers on quitting smoking and, among those who continued to smoke, on reduction of cigarettes per day from baseline to the latest follow-up available. To do so, we ran univariate and multivariate models entering the covariates tested in step 1.

## Results

### Sample Description

Proportion of the sample enrolled by year was: 2009 (4%), 2010 (36%), 2011 (24%), 2012 (21%), and 2013 (15%). The sample (N = 956) was 50% men, with mean age 39 (SD = 14), and 21% employed; 15% were Hispanic and 57% Caucasian, 24% African American, 5% Asian/Pacific Islander, and 14% multiracial/other. Psychiatric diagnoses were 27% unipolar depression, 32% bipolar depression, and 27% nonaffective psychotic disorder; participants not meeting criteria for one of these major groups were classified as other (14%). In addition, 68% of participants met criteria for alcohol or illicit drug abuse or dependence. Most (80%) had been hospitalized for psychiatric treatment previously, with a median of 4 hospitalizations (IQR: 2, 10). The sample's mean BASIS-24 summary score (M = 2.1, SD = 0.8) indicated greater severity than published norms for hospitalized psychiatric samples [44], and the mental health component score on the SF-12 averaged two standard deviations lower (more severe) than published norms (M = 31, SD = 14) [43].

In terms of tobacco use, prior to hospitalization, participants averaged 17 (SD = 10) cigarettes per day for 19 (SD = 14) years, 78.5% smoked within 30 minutes of waking. Stage of change for quitting smoking was 29.6% precontemplation, 46.8% contemplation, and 23.6% preparation.

### EC Use by Time

Among N = 956 smokers with SMI, 101 reported EC use (11%). The proportion of the sample reporting EC use by year of study enrollment was 0% (0 of 35) in 2009, 1% (5 of 348) in 2010, 9% (21 of 225) in 2011, 19% (38 of 202) in 2012, and 25% (37 of 146) in 2013. By year, EC use was not reported prior to 2011; among those reporting trying EC, 7% first reported use in 2011, 21% in 2012, and 72% in 2013 or early 2014.

### Correlates of EC Use

We ran a logistic regression model to examine study factors (condition, year of enrollment, hospital site), demographic (gender, Hispanic ethnicity, racial group, age group, employment status), psychiatric (diagnosis, substance use disorder, SF-12), and tobacco-related variables (usual cigarettes per day at baseline, TTFC, stage of change) associated with EC use. Due to concerns with multicollinearity, age (and not years of smoking) and the SF-12 Mental Health Component Score (and not the BASIS-24 summary score) were entered into the model. We dichotomized the SF-12 mental health component score at 2 or more standard deviations below the published norm value of 50 (i.e., score <30).

Table 1 presents the results of the multiple logistic regression. With all variables in the model, EC use was significantly associated with year of study entry, participant age, Hispanic ethnicity, and smoking stage of change. The odds of EC use increased with each additional year of study enrollment. By age, young adults had the highest reported use of EC, while adults age 36–45 had the lowest. Also associated with lower likelihood of EC use was identifying as Hispanic. The only smoking variable associated with EC use was stage of change. Relative to smokers in precontemplation, those in preparation had more than twofold greater likelihood of reporting EC use. Treating hospital, intervention condition, race, gender, psychiatric diagnosis, substance use disorder, mental health functioning, and severity of tobacco use (cigarettes per day, TTFC) were unrelated to EC use.

**Table 1:** Descriptive Characteristics and Multiple Logistic Regression Model Testing Study-level, Demographic, Psychiatric, and Tobacco-Related Variables Associated with E-Cigarette Use.

	n	% Reporting Recent E-cigarette Use	OR	95% CI for OR	
				Lower	Upper
Year enrolled in study					
2009–2010 (ref)	383	1%	-	-	-
2011	225	9%	7.78*	2.84	21. 92
2012	202	19%	19.43*	7.33	51.49
2013	146	25%	29.15*	10.53	80.72
Condition					
Control group (ref)	134	10%	-	-	-
Brief treatment	414	9%	1.01	.49	2.07
Extended treatment	408	11%	1.40	.68	2.85
Hospital site					
Hospital 1 (ref)	192	9%	-	-	-
Hospital 2	724	10%	1.33	.70	2.51
Hospital 3∧	40	23%	1.33	.46	3.81
Gender					
Men	482	11%	1.26	.78	2.03
Women (ref)	474	9%	-	-	-
Employment status					
Employed	204	11%	1.15	.65	2.01
Unemployed (ref)	752	10%	-	-	-
Age group					
18–25	225	17%	2.61*	1.19	5.72
26–35	189	11%	1.07	.47	2.44
36–45	207	4%	.41	.16	1.05
46–55	214	8%	.93	.40	2.15
56+ (ref)	121	9%	-	-	-
Ethnicity					
Hispanic (ref)	141	6%	-	-	-
Non-Hispanic	814	11%	4.00*	1.81	8.86
Race					
Caucasian (ref)	454	11%	-	-	-
African American	218	6%	.57	.29	1.10
Asian/Pacific Islander	44	10%	.87	.30	2.50
Multiracial/other	145	11%	1.00	.50	1.98
Diagnosis					
Psychosis (ref)	255	9%	-	-	-
Unipolar	258	11%	1.12	.57	2.17
Bipolar	304	11%	1.16	.62	2.16
Other	139	8%	.80	.35	1.85
Alcohol/drug use disorder					
Yes	645	11.2%	1.30	.76	2.21
No (ref)	311	9.3%	-	-	-
Mental health functioning∨					
>2 SD below norms (ref)	474	10.8%	-	-	-
≤2 SD below norms	461	10.6%	.89	.55	1.44
Stage of change					
Precontemplation (ref)	282	8%	-	-	-
Contemplation	448	10%	1.56	.86	2.83
Preparation	226	13%	2.68*	1.38	5.20
First cigarette in AM					
Within 30 min	204	10%	1.40	.76	2.57
>30 min (ref)	746	9%	-	-	-
Baseline cigarettes/day					
1–19 per day (ref)	544	10%	-	-	-
20+ per day	403	10%	1.39	.84	2.29

OR  =  odds ratio, CI  =  confidence interval.

* p<.05 for group comparison.

^∧^Hospital added as a recruitment site in 2013.

^∨^SF-12 Mental health component score categorized as functioning below the normative value of 50 by more than 2 standard deviations (i.e., scores below 30).

### EC Use and Change in Conventional Cigarette Use

Though follow-up data are still being collected for the clinical trial, analysis of smoking status at the latest follow-up available indicated that 21% of those reporting EC use and 19% of participants not reporting EC use were tobacco abstinent at their latest follow-up assessment available, X^2^ = 0.12, p = .726. Among those who continued to smoke, EC users did not report significantly greater reduction in cigarettes per day from baseline to latest available follow-up (mean reduction of −7.1 cigarettes/day (SD = 12.5) for EC vs. −6.6 (SD = 11.0) for non-EC users, F_(1,703)_ = .12, p = .730) nor lighter daily consumption of cigarettes at the latest follow-up available (mean of 10.0 cigarettes/day (SD = 8.9) for EC vs. 10.1 (SD = 9.0) for non-EC, F_(1,710)_ = .01, p = .915). Analyzed as a 50% reduction also indicated no difference by group: 51% of both EC and non-EC users reported reducing by half or more their number of cigarettes smoked per day from baseline to longest follow-up available, X^2^ = .001, p = .978. In multivariate models adjusting for the sample characteristics listed in Table 1, e-cigarettes remained unrelated to abstinence status, and among those who continued to smoke, unrelated to reduction in cigarettes per day (analyzed continuously or as a 50%+ reduction) and cigarette per day consumption at latest follow-up available (all p's>.550).

## Discussion

The place of EC in smoking cessation is currently one of the leading clinical, public health, and regulatory policy issues in the field. The significant disparities in tobacco use and adverse health effects among persons with SMI call for their inclusion in scientific investigation of EC uptake and use for cessation and harm reduction.

Like smokers surveyed in the general population, in the current trial, smokers with SMI were increasingly using EC over time, particularly young adults (age 18–25). In contrast, participants identifying as Hispanic reported a lower likelihood of EC experimentation. EC use did not vary by gender, race, employment status, geography (hospital site), treatment condition, psychiatric or substance use diagnosis, or level of mental health functioning.

Whereas general population-based surveys have reported mixed findings on smokers' EC use and readiness to quit tobacco, our study found smokers preparing to quit in the next 30 days had a more than twofold greater likelihood of EC use [25], [45]–[47]. Involvement in a study on smoking and exposure to study treatments may have enhanced receptivity to advertising and popular messaging about use of EC for smoking cessation among those ready to quit. As a signal for clinicians, patients' reports of EC use may indicate readiness to quit and present opportunities to encourage and support quit attempts with evidence-based treatments such as NRT and behavioral counseling. Unrelated to EC use, were baseline measures of tobacco dependence severity, measured by usual number of cigarettes smoked per day prior to hospitalization and time to first cigarette smoked in the morning.

Notably, study participants reporting EC use did not differ during the prospective trial in their abstinence status nor, among those who did not quit, reduction in smoking, consistent with recently published findings in the literature [25], [48]. For example, in a longitudinal analysis of data from a general population of smokers, past 30-day EC use was not a significant predictor of quitting or reduction in cigarette consumption at one-year follow-up [48]. In a longitudinal study of US quitline callers, EC users had significantly lower quit rates at 7-months follow-up compared to non-users [49]. Taken together with the present findings, it appears that those who try EC are not more likely to reduce their tobacco cigarette consumption or increase their success with quitting.

A few participants reported encouragement to use EC by their clinicians, who suggested EC as a healthier alternative to conventional cigarettes. Given the lack of evidence that EC use supports cessation or reduction in smoking, the findings highlight the urgent need for research, training, and evidence-based clinical practice recommendations pertaining to EC use. Further, with the rapidly evolving tobacco marketplace, patient assessments ought to be broadened to capture nicotine and tobacco products beyond conventional cigarettes. Case in point, we did not anticipate the future growth of EC at the time our trial was initiated in 2009. Given technological advances and the tobacco industry's efforts to retain its market share, a broad-based assessment of “other tobacco products” was helpful for monitoring use of emerging products. Regarding the question of permitting or even providing EC in hospital settings, evaluation and consideration is needed of aerosol contents and secondhand exposure risks as well as the consequences of re-normalizing cigarette use in medical/treatment settings.

Observed within a large, longitudinal clinical trial of smokers with SMI, reporting and detection of EC use increased over time. While our indirect assessment likely underestimated actual use in the sample and did not assess duration and amount of use, the observed increase is consistent with greater EC awareness and use in the general population [28]. The observed trend is of clinical interest and a signal warranting further epidemiologic and treatment research.
